# Oral Anticoagulation Therapy in Heart Failure Patients in Sinus Rhythm: A Systematic Review and Meta-Analysis

**DOI:** 10.1371/journal.pone.0052952

**Published:** 2013-01-02

**Authors:** Giuseppe Rengo, Gennaro Pagano, Alessandro Squizzato, Lorenzo Moja, Grazia Daniela Femminella, Claudio de Lucia, Klara Komici, Valentina Parisi, Gianluigi Savarese, Nicola Ferrara, Pasquale Perrone-Filardi, Dario Leosco

**Affiliations:** 1 Division of Cardiology, “Salvatore Maugeri” Foundation, IRCCS – Scientific Institute of Telese Terme, Benevento, Italy; 2 Department of Medical Translational Sciences, Federico II University of Naples, Naples, Italy; 3 Research Center on Thromboembolic Disorders and Antithrombotic Therapies, Department of Clinical and Experimental Medicine, University of Insubria, Varese, Italy; 4 Department of Biomedical Sciences for Health, University of Milan, Milan, Italy; 5 IRCCS Galeazzi Orthopedic Institute, Milan, Italy; 6 Department of Advanced Biomedical Sciences, Federico II University of Naples, Naples, Italy; Universidad Peruana de Ciencias Aplicadas (UPC), Peru

## Abstract

**Background:**

Heart failure (HF) patients show high morbidity and mortality rate with increased risk of malignant arrhythmia and thromboembolism. Anticoagulation reduces embolic event and death rates in HF patients with atrial fibrillation, but if antithrombotic therapy is beneficial in patients with HF in sinus rhythm is still debated.

**Methodology and Principal Findings:**

We conducted a systematic review of prospective, randomized controlled trials (RCTs) to assess the efficacy and safety of oral anticoagulant therapies (OATs) compared to antiplatelet treatment in HF patients in sinus rhythm. MEDLINE, Web of Science, CENTRAL and Scopus databases were searched up to May 2012. Four RCTs were identified and a total of 3663 patients were included in the meta-analysis. Patients with both ischemic and non-ischemic HF were included. There was no significant difference in mortality (odds ratio (OR) 1.01, 95% confidence interval (CI) 0.86 to 1.19) between OATs group and antiplatelet drug group. OATs have reduced ischemic stroke risk (OR 0.49, 95% CI 0.32 to 0.74), but have increased major bleeding risk (OR 2.01, 95% CI 1.40 to 2.88) compared to antiplatelet treatment.

**Conclusion:**

In HF patients in sinus rhythm OATs do not show a better risk-benefit profile compared to antiplatelet treatment in cardioembolism prevention. Warfarin and aspirin seem to be similar in reducing mortality. Warfarin reduces the incidence of ischemic stroke, but increases major bleedings. Thus, it is possible to speculate that aspirin prescription be indicated in patients with high risk of bleeding, whereas warfarin could be preferred in patients with high thromboembolic risk.

## Introduction

Heart failure (HF) is a growing public health problem worldwide, and it is associated with an increased risk of left ventricular thrombus formation and cerebral embolism due to endothelial dysfunction, reduced blood flow and underlying state of hypercoagulability [1]–[4]. In the population-based Framingham Heart Study, the relative risk of stroke in individuals with HF compared to those without HF was 4.1 for men and 2.8 for women [5]. The risk of cardioembolism is further enhanced by the presence of atrial fibrillation (AF), however HF patients in sinus rhythm still have higher thromboembolic risk. A retrospective analyses reports a yearly incidence of thromboembolism of 1.0%–4.5% in HF patients without AF [6]. In the SAVE study, an observational analysis of 2231 patients with left ventricular dysfunction after acute myocardial infarction, 4.6% of patients had fatal or non fatal strokes during the study period (rate of stroke per year of follow-up, 1.5 percent) and the estimated five-year stroke rate was 8.1 percent in the entire population [7], [8]. Antiplatelet therapy is commonly prescribed in HF patients in sinus rhythm since ischemic cardiomyopathy is the principal underlying cause [9], [10]. Conversely, oral anticoagulant therapy (OAT), that includes oral vitamin K antagonists (VKAs) and new oral anticoagulant therapies, is commonly prescribed in HF patients with AF since it has been shown more efficacious than aspirin in reducing embolic risk [9], [11]. International guidelines recommend the use of VKAs in HF patients with AF to prevent cardioembolic risk but OAT is not indicated in HF patients without AF [12], [13], [14].

The aim of the present meta-analysis has been to assess the efficacy and safety of OAT in comparison to antiplatelet treatment in HF patients in sinus rhythm.

## Methods

The study was designed according to the PRISMA (Preferred Reporting Items for Systematic Reviews and Meta-Analyses) statement [15].

### Search Strategy

MEDLINE, Web of Science, Cochrane CENTRAL, Scopus databases were searched for articles in all languages published until May 2012. Gray literature was not considered as a priority asset of our systematic review. Studies were identified and evaluated by the authors (GR, GP, AS) using the major medical subject heading combined with text and key words. As example for MEDLINE (“heart failure”[MeSH Terms] OR “heart failure”[All Fields] OR (“heart”[All Fields] AND “failure”[All Fields])) AND (“anticoagulants”[MeSH Terms] OR “anticoagulants”[All Fields] OR (“anti”[All Fields] AND “coagulant”[All Fields]) OR “anti coagulant”[All Fields] OR “warfarin”[MeSH Terms] OR “warfarin”[All Fields] OR “antithrombins”[MeSH Terms] OR “antithrombins”[All Fields] OR “antithrombin”[All Fields] OR “aspirin”[MeSH Terms] OR “aspirin”[All Fields]). Additional eligible studies were identified screening the reference lists of studies included in our analysis.

### Study Selection

All selected titles and abstracts were independently reviewed by two authors (GP,GR). Studies were excluded if the title and/or abstract were not appropriate for the aim of our review. Full texts were subsequently obtained for eligible studies or when the relevance of an article could not be certainty excluded. Disagreement was resolved by consensus and by opinion of a third reviewer (AS), when necessary. Selected studies were eligible if they met the following criteria: patients with heart failure due to any underlying cause without AF; adults only; patients treated with OAT or antiplatelet treatment; at least 100 patients enrolled; duration of treatment at least 1 month; RCT design. Reviews, case-reports, non-human studies and abstracts or conference proceedings were excluded. In summary, the present meta-analysis included only RCTs that compared the efficacy and the safety of OAT versus antiplatelet treatment among HF patients in sinus rhythm.

### Risk of Bias in Included Studies

Using the guidelines in the Cochrane Handbook for assessment of risk of bias (RoB), RCTs were graded by two independent reviewers (GR and GP) basing on sequence generation, allocation concealment, incomplete outcome data, selective outcome reporting, blinding of participants and personnel and blinding of outcome assessment [16]. These items were considered as key domains for RoB assessment and classified as “adequate” (low risk of bias), “inadequate” (high risk of bias), or “unclear”. Studies with adequate procedures in all domains were considered to have a low risk of bias; ones with inadequate procedures in one or more key domain(s) were considered to have a high risk of bias; and ones with unclear procedures in one or more key domain(s) were considered to have an unclear risk of bias. Disagreement was resolved by consensus and by opinion of a third reviewer (AS).

### Data Extraction and Types of Outcomes Measures

Data extraction has been completed by two reviewers (GR, GP) independently using a standardized form. Disagreement was resolved by consensus and by the opinion of a third reviewer (LM), when necessary. Overall mortality was the primary outcome. Additional efficacy outcomes were: ischemic stroke, myocardial infarction (MI), hospital admission. Main safety outcomes were: major bleedings and intracerebral bleedings. A separated analysis was planned for HF patients with ischemic cardiomyopathy and with non-ischemic cardiomyopathy. To define the severity of bleeding events we planned to use the International Society of Thrombosis and Haemostasis (ISTH) classification [17].

### Statistical Analysis

Odds ratios (OR) and 95% confidence intervals (CI) were calculated. The results were pooled using the inverse variance method. The random effect model described by DerSimonian and Laird [18] was used to synthesize data rather than the fixed effect model because it incorporates intra- and inter-study variability. The software Review Manager (RevMan, version 5.1.4 for Windows 7, Copenhagen: The Nordic Cochrane Centre, The Cochrane Collaboration, 2011) supported the analysis.

### Assessment of Heterogeneity and Publication Bias

Heterogeneity was assessed using I^2^ statistic that accounts of between-study (or inter-study) variability as opposed to within-study (or intra-study) variability. Because of latent clinical heterogeneity, random-effects model was used, independently of statistical evidence for heterogeneity. Heterogeneity has been considered substantial if I^2^ value was greater than 25% [19]. If substantial heterogeneity was identified, subgroup analyses and sensitivity analyses were performed. Presence of publication bias was explored performing the test for asymmetry of the funnel plot by Egger that is a linear regression of normalized effect estimate (estimate divided by its standard error) against precision (reciprocal of the standard error of the estimate) [20], [21].

## Results

**Figure 1:**
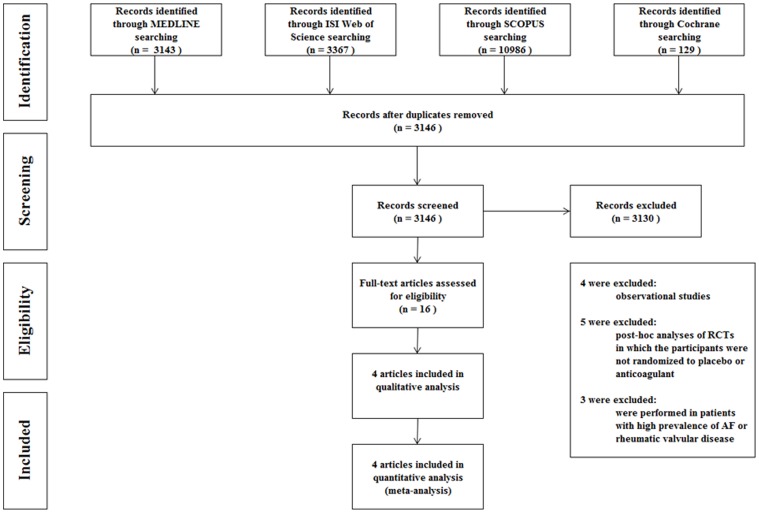
Meta-analysis flow chart.

**Figure 2:**
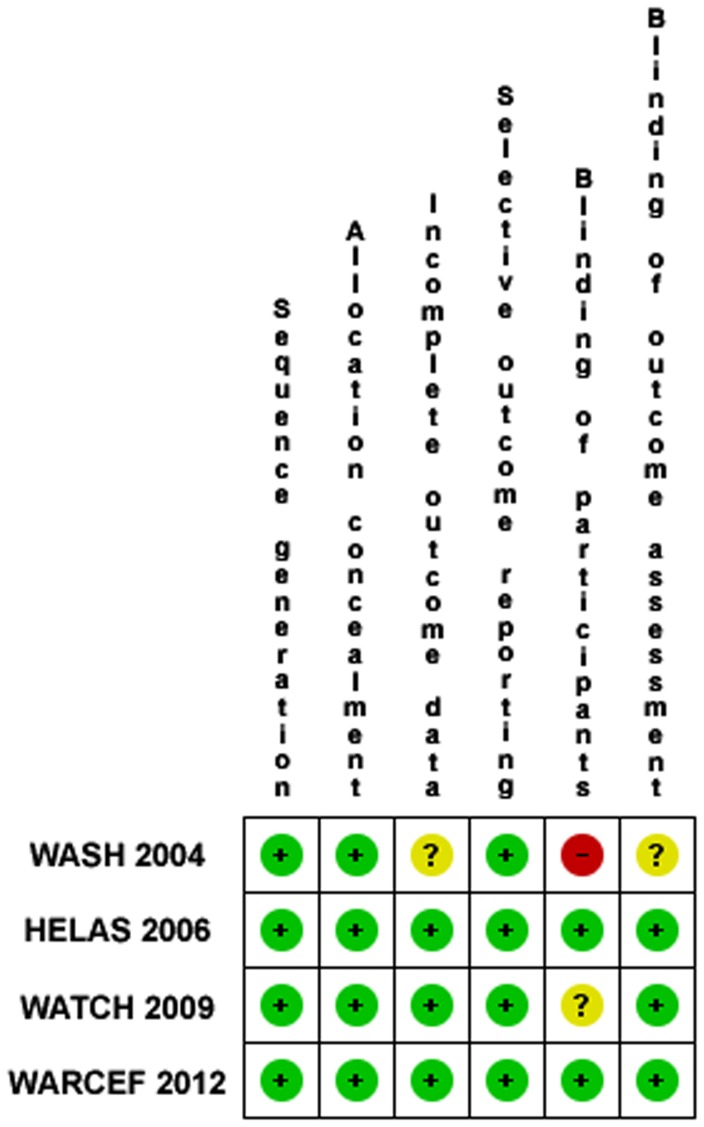
Risk of bias summary: review authors’ judgment about each risk of bias item for each included study.

Of 17625 articles identified by the initial search, 16 were retrieved for more detailed evaluation and 4 were finally included in the meta-analysis (Figure 1) [22]–[25]. All included studies have enrolled patients without atrial fibrillation However, we found three studies not reporting separated data for patients in sinus rhythm and in atrial fibrillation, thus they were excluded from meta-analysis [26]–[28]. Baseline characteristics of patients included in the studies were summarized in Table 1. Studies population sizes ranged from 180 [22] to 2305 [25] patients, for a total of 3663 included patients. Quality assessment items have been summarized in Figure 2. The WASH study [22], the pilot study of WATCH study, included 279 patients that were randomized to warfarin (target INR 2.5), aspirin (325 mg), or placebo and followed up for a mean of 27 months. The HELAS trial [23] separated 197 patients according to the etiology of their HF. Only patients in the ischemic cardiomyopathy group (n.115) were randomized to receive warfarin (target INR 2–3) or aspirin (325 mg), while patients in the non-ischemic group (n.82) were randomized to receive warfarin or placebo. Patients were followed up for a mean period of 20 months. We included in our meta-analysis only the ischemic group since it compared warfarin versus aspirin. The WATCH trial [24] included 1587 HF patients receiving aspirin (162 mg), clopidogrel (75 mg), or warfarin (target INR 2-3.5), followed up for a mean duration of 21 months. The WARCEF trial [25] randomized 2305 HF patients to receive aspirin (325 mg) or warfarin (target INR 2.5–3.5), with a mean follow up of 42 months.

**Table 1:** Characteristics of studies.

Article	Trialacronym	Year	FU(m)	Primary efficacyoutcome	Safetyoutcome	Major bleedingdefinition	Treatment	Control	Treat.(n)	Ctrl(n)	Pts(n)	Age(yrs)	Male(%)	NYHAclass	LVEF(%)
Cleland	WASH	2004	27	Death, non-fatal MI,non-fatal stroke	Major hemorrhage	Hemorrhage requiring blood transfusion.	Warfarin(INR 2.5)	Aspirin 300 mg	89	91	180	63	75	III–IV	NA
Cokkinos	HELAS	2006	20	Death, pulmonaryembolism, non-fatalstroke, hospitalization,exacerbation of HF	Cerebrovascular events ascribed to intracranial haemorrhage andbleeding while onstudy drug	NA	Warfarin(INR 2–3)	Aspirin 325 mg	54	61	115	62	90	II–IV	29
Massie	WATCH	2009	21	Death, non-fatal MI,non-fatal stroke	Major bleedings	Bleedings leading to death or disability, requiring surgical intervention, or trasfusion or associated with an acute declineof hemoglobin >2 g/dl	Warfarin(INR 2–3.5)	Aspirin 162 mg	540	523	1063	63	85	II–IV	25
Homma	WARCEF	2012	42	All Death and death dueto ischemic stroke orintracerebral bleedings	Intracerebral bleedingsor intracranialhemorrhage	Major hemorrhage was defined as intracerebral, or retinal hemorrhage; bleeding causing a decline in the hemoglobin level of more than 2 g; or bleeding requiring transfusion, hospitalization, or surgical intervention.	Warfarin(INR 2.5–3.5)	Aspirin 325 mg	1142	1163	2305	61	80	I–IV	25

FU, Follow-up; m, months; EF, left ventricular ejection fraction; NA, not available.

### Efficacy Outcomes

**Figure 3:**
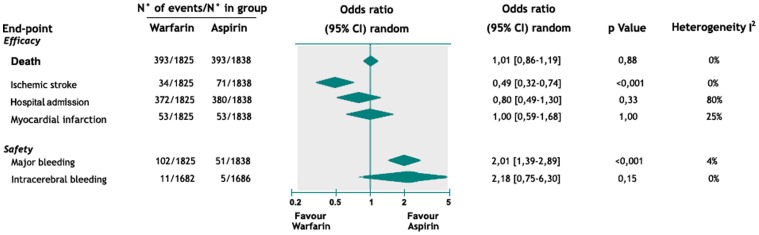
Pooled event rate and odds risk ratio for major end point in overall cohort patients with heart failure in sinus rhythm.

In the cumulative analysis of all patients (n = 3663), mortality rate was not significantly different between warfarin and aspirin groups (OR 1.01, 95% CI 0,86 to 1,19; I^2^ = 0%). Warfarin was associated with a significant reduction of ischemic stroke compared to aspirin (OR 0.49, 95% CI 0.32 to 0.74; I^2^ = 0%, NNT = 50). Hospital admission and MI rates were not significantly different between warfarin and aspirin groups (OR 0.80, 95% CI 0.49 to 1.30, I^2^ = 80% and OR 1.00, 95% CI 0.59 to 1.68; I^2^ = 25% respectively) (Figure 3). One study removed analysis showed that, only when the WARCEF study was excluded from analysis, there was a reduced hospital admission rate in OAT compared to aspirin group with a statistically significant reduction in heterogeneity (OR = 0.65, C.I. 0.50 to 0.85; I^2^ = 0%, NNT = 15). Subgroup analyses in ischemic and non ischemic patients were not performed since only one study provided separated data for HF etiology [23].

### Safety Outcomes

Data about bleeding events were not reported in enough trials for applying ISTH classification and the definition of major bleeding provided by authors was used. In the overall cohort of patients (n = 3663), OAT was associated with significant increase in major bleeding events compared to aspirin (OR 2.01, 95% CI 1.39 to 2.89; I^2^ = 4%, NNH = 35). Moreover, a trend to increase in intracerebral bleedings has been observed in the warfarin group, but it was not statistically significant (OR 2.18, 95% CI 0.75 to 6.30; I^2^ = 0%).

### Publication Bias

No publication bias was detected by Egger's linear regression method for each single outcomes analysis although the limited number of studies could not rule out a possible publication bias (data not shown).

## Discussion

In the medical community there is not general consensus in either recommending or advising against OAT in HF. Several evidence have recommended OAT in HF patients with AF, as prevention for the high cardioembolic risk observed in this population. Differently, the majority of HF patients due to ischemic etiology takes aspirin for secondary prevention of coronary heart disease. Therefore, the aim of this present meta-analysis has been to assess potential differences in efficacy and safety between these two therapeutic modalities in HF patients in sinus rhythm. Our results indicate that warfarin does not show a better efficacy-safety profile compared to aspirin in preventing cardioembolism in HF patients without AF. However, when compared to aspirin, warfarin was associated with a significant reduction (OR 0.49) of ischemic stroke incidence with a relative risk reduction that is comparable to that reported in HF patients with AF treated with OAT [29]. Nevertheless, due to the low annual stroke rate observed in HF patients in sinus rhythm (between 0.8% and 3.2% per year), the advantages deriving from routine anticoagulation cannot overcome the increased risk of bleedings related to warfarin use [24], [30]–[33]. Consistently, our data show that OAT was associated with a more than doubled bleeding risk, with a trend to increase also the risk of intracerebral bleedings. It is important to underline, that only in the HELAS study, HF patients were dived for ischemic or non ischemic cardiomyopathy, thus no conclusions can be drown about efficacy or safety in these different patient subpopulations.

In HF patients with non ischemic etiology and without AF, no studies are available about efficacy and safety of OAT or antiplatelet therapy compared to placebo, whereas several line of evidence support the use of aspirin in HF patients due to ischemic etiology as secondary prevention of coronary artery disease. Thus, RCTs comparing aspirin vs. placebo in HF patients in sinus rhythm and non ischemic etiology might be helpful to guide therapy in this specific HF subpopulation. In the present analysis the hospital admission rate for worsening HF was lower in warfarin group only after exclusion of WARCEF study. Its exclusion reduces the heterogeneity among studies but unfairly reduces the available information since this is the largest trial with the longest follow-up. The important issue regarding discordant results on the incidence of hospital admissions for worsening HF in patients treated with warfarin or aspirin is not addressable. Unfortunately, despite it would be of great interest whether warfarin treatment impacts quality of life (especially in terms of anxiety burden related to OAT monitoring), the identified trials did not investigate this relevant aspect which certainly would be an important argument for future trial investigations.

### Strengths and Limitations of this Meta-analysis

The main strengths of our review include the systematic strategy and the high score at Cochrane quality assessment for all trials included. Our meta-analysis has one major shortcoming. It was not carried out on individual patients data exploring subgroups with higher thromboembolic risk. Moreover, there are some limitations in the outcome evaluation due to the too small sample size and the too short follow up (i.e. 2 years) period, resulting in a low number of events in the trials included. The exclusion of gray literature could be a limitation of our search strategy. It is important to underline that in WATCH trial, aspirin dosage is lower than that used in the other trials, thus explaining the lower efficacy of treatment reported. Although differences in trial definitions of outcomes among different trials should be considered for the interpretation of the overall result, it is important to mention that mortality and ischemic stroke, the principal efficacy outcomes evaluated in our study, are hard endpoint not affected by study definitions. The absence of heterogeneity in the most part of analysis supports the strength of our results. Further studies are needed to better identify high risk HF subgroups. The definition of an HF risk stratification score, similar to that available for ischemic risk assessment (CHADS2 or CHA2DS2-VASc scores), or bleeding risk assessment (HAS-BLED) [34], [35], will be useful to identify HF patients at higher risk. Finally, no evidences are provided regarding the use of new oral anticoagulants (oral direct thrombin inhibitors, oral Factor Xa inhibitors) which seem to offer a different risk–benefit profile compared to warfarin and might induce a reduction in ischemic stroke rates with less risk of major bleeding. Thus, an head to head comparison between warfarin and new anticoagulants (rivaroxaban, apixaban and dabigatran), with antiplatelet therapy might be of great interest in HF patients in sinus rhythm.

### Conclusions

In patients with HF in sinus rhythm, warfarin and aspirin seem to be similar in reducing mortality. Warfarin reduces the incidence of ischemic stroke, but increases major bleedings. Thus, it is possible to speculate that aspirin could be indicated in patients with high risk of bleeding, whereas warfarin could be preferred in patients with high thromboembolic risk. However, further studies are needed to clarify the role of antitrombotic therapy in HF patients in sinus rhythm, particularly in the subpopulation with non ischemic etiology.
